# Multiple Regulations of Parasitic Protozoan Viruses: A Double-Edged Sword for Protozoa

**DOI:** 10.1128/mbio.02642-22

**Published:** 2023-01-12

**Authors:** Zhiteng Zhao, Xin Li, Nan Zhang, Jianhua Li, Na Zhao, Mengyao Gao, Xichen Zhang, Xiaocen Wang, Panpan Zhao, Lu Li, Min Sun, Lili Cao, Pengtao Gong

**Affiliations:** a State Key Laboratory for Zoonotic Diseases, College of Veterinary Medicine, Jilin University, Changchun, China; b Faculty of Life Science, Thangshan Normal University, Thangshan, China; c Jilin Academy of Animal Husbandry and Veterinary Medicine, Changchun, China; Albert Einstein College of Medicine

**Keywords:** parasitic protozoan viruses, PPVs, morphology, pathogenicity, viral infection, virus-parasite relationship

## Abstract

Parasite infections affect human and animal health significantly and contribute to a major burden on the global economy. Parasitic protozoan viruses (PPVs) affect the protozoan parasites’ morphology, phenotypes, pathogenicity, and growth rates. This discovery provides an opportunity to develop a novel preventive and therapeutic strategy for parasitic protozoan diseases (PPDs). Currently, there is greater awareness regarding PPVs; however, knowledge of viruses and their associations with host diseases remains limited. Parasite-host interactions become more complex owing to PPVs; however, few studies have investigated underlying viral regulatory mechanisms in parasites. In this study, we reviewed relevant studies to identify studies that investigated PPV development and life cycles, the triangular association between viruses, parasites, and hosts, and the effects of viruses on protozoan pathogenicity. This study highlights that viruses can alter parasite biology, and viral infection of parasites may exacerbate the adverse effects of virus-containing parasites on hosts or reduce parasite virulence. PPVs should be considered in the prevention of parasitic epidemics and outbreaks, although their effects on the host and the complexity of the triangular association between PPVs, protozoans, and hosts remain unclear.

## INTRODUCTION

The parasite-host relationship is a symbiotic and not mutually beneficial association, which produces detrimental effects on the host (1). Viruses can infect and replicate within single-celled protozoa, a phenomenon referred to as virus parasitism, a parasitic process commonly known as hyperparasitism. Viruses that are exclusively parasitic on parasitic protozoa are known as parasitic protozoan viruses (PPVs). From early studies to current discoveries, virus-like particles (VLPs) have been found in various parasitic protozoans, including Entamoeba histolytica (2) and *Leishmania hertigi* (3), among others. Initially, protozoan viruses, such as *Totivirus*, *Victorivirus*, *Giardiavirus*, and *Leishmaniavirus*, were considered to belong to the *Totiviridae* family. However, the identification of numerous protozoan viruses has refuted this classification. Some protozoan viruses, such as E. histolytica, which may have a negative single-stranded RNA (ssRNA) genome, belong to the *Rhabdoviridae* family (4). Additionally, the Acanthamoeba polyphaga virus (*Mimivirus*) has double-stranded DNA (dsDNA) (5), which remains unexplained.

Notably, dsRNA/ssRNA protozoan viruses, such as the Trichomonas vaginalis virus (TVV) (6), Giardia lamblia virus (GLV) (7), Cryptosporidium parvum virus 1 (CSpV1) (8) appear as non-enveloped, non-segmented, primarily spherical icosahedral virion particles when viewed using transmission electron microscopy (TEM). Molecular biological techniques (such as PCR or metagenomic analysis) are used to identify the protozoan virus genome, which ranges from 4 to 7 kb and has a diameter of 30 to 40 nm. Two proteins, the capsid protein (CP) and RNA-dependent RNA polymerase (RdRp) are individually encoded by the viral genome.

An intriguing subject of current research is the effect of PPVs on the parasite itself or the host-parasite association (1). Some PPVs regulate the biological characteristics of the parasite itself, such as the parasite proliferation rate and metabolic processes (gluconeogenesis and lipid metabolism) (9). Others may regulate parasite-host interactions, affect the infection mode and pathogenesis, and attenuate or delay the recognition and clearance from the host immune system (10), which therefore makes PPVs allies of the parasite. Additionally, PPVs can use parasites as vectors to protect themselves from adverse environmental effects (5, 11). More and more studies support the theory that viral infections modify parasite-host interactions. This article will serve as a valuable reference for future research in parasitic infections, and provide new avenues and guidelines for protozoan disease detection and prevention, as well as research on protozoa-host associations.

## DISCOVERY OF PARASITIC PROTOZOAN VIRUSES

Following advances in TEM and molecular biology techniques, a variety of PPVs continue to be discovered. The TVV was the first protozoan virus that was characterized biochemically (12); however, its virion protein complex has not been clearly defined as a single viral species (13, 14). Based on phylogenetic distinctions, at least 4 major TVV species have been identified in the *Trichomonasvirus* strain, with TVV1 being closer to TVV2 and TVV3 than to TVV4 (15). The fifth species belonging to the *Trichomonasvirus* strain was named TVV5 and was recently characterized by a novel sequence assembly and classification; TVV5 shows low sequence identity with the other 4 species (16).

GLV was initially detected in a Portland I strain and subsequently in other assemblages (7, 17). In addition, high-throughput sequencing of GLVs from different isolates (18) identified a new unclassified viral sequence (GdRV-2), which has high homology to *Totiviridae* and *Botybirnaviridae* but is unrelated to the *Giardiavirus* (18).

The *Leishmania* RNA virus (LRV), first discovered in *L. hertigi* promastigotes (3) has 2 subgenera, LRV1 and LRV2, with low similarity (<40%) (19, 20). They separately infect the subgenus *Viannia* (*L. braziliensis*, *L. guyanensis*, *L. lainsoni*, *L. naiffi*, L. panamensis, and *L. shawi*) and the subgenus *Leishmania* (*L. aethiopica*, L. infantum, L. major, and *L. tropica*) (21). *Leishbunyavirus* (LBV, also referred to as the LmarLBV1 strain) is a novel non-LRV RNA virus that was first identified in *L. martiniquensis* (a member of the *Leishbuviridae* family) (22). The virion is a negative-sense single-stranded RNA (23).

In 1997, two dsRNA viral-associated nucleic acids were first identified within the C. parvum cytoplasm (8). The morphological characteristics and the replication mechanism of *Cryspovirus* are similar to those of the *Partitiviridae* family (24). *Cryspovirus* has been detected in C. hominis, C. felis and *C. meleagridis* (25). Various PPV types have been identified in other species including *Babesia*, *Eimeria* and other protozoa (26–29) (Table 1 and 2).

**TABLE 1:** Summary of the main characteristics of the viruses found in protozoan parasites

Virus	Family/genus	Pathogen	Virion (shape/diam)	Genome	Genome size	ORFs	Earliest discovery time
Entamoeba histolytica virus	unassigned	Entamoeba histolytica	Polyhedral/70 nm	/	/	/	1972
LRV1	*Totiviridae/Leishmaniavirus*	*L. guyanensis*	Icosahedral/ ~40 nm	Monopartite, linear dsRNA	~5.3 kb	CP and CP/RdRp	1974
		*L. shawi*					
		*L. brasiliensis*					
TVV1–4	*Totiviridae/Trichomonasvirus*	T. vaginalis	Icosahedral/ ~33 nm	Monopartite, linear dsRNA	~4.5 to 5 kb	CP and CP/RdRp (2 partially overlapping)	1985
GLV	*Totiviridae/Giardiavirus*	G. duodenalis	Icosahedral/ ~36 nm	Monopartite, linear dsRNA	~6.3 kb	CP and CP/RdRp (2 partially overlapping)	1986
/^*a*^	*/*	Babesia bovis	~38 nm	linear dsRNA	~5.5 kb	/	1991
LRV2	*Totiviridae/Leishmaniavirus*	L. major	Icosahedral/ ~40 nm	Monopartite, linear dsRNA	~5.2 kb	CP and CP/RdRp	1995
		*L. tropica*					
		*L. aethiopica*					
		L. infantum					
CSpV1	*Partitiviridae/Cryspovirus*	C. parvum	Icosahedral/~31 nm	Bi-segmented, linear dsRNA	dsRNA1: ~1.8 kb	dsRNA1: RdRp	1997
		C. hominis			dsRNA2: ~1.4 kb	dsRNA2: CP	
		C. felis					
		*C. meleagridis*					
Bcvir	*/*	*Babesia canis*	/	dsRNA	~2.8 kb	ORF1(~426 bp) and ORF2 (~503 bp) (2 partially overlapping)	2003
APMV	*Mimiviridae/unassigned*	Acanthamoeba polyphaga	Icosahedral/~400 nm	Monopartite, circular dsDNA	~800 kb	21 ORFs	2003
MaRNAV1	*unassigned/narnalikevirus*	P. vivax	No virion	Bi-segmented, linear ssRNA (+)	segment I: ~2.9 kb segment II: ~2.6 kb	segment I: RdRp segment II: CP	2019
LmarLBV1	*putative new genus/Leishbunyavirus*	*L. martiniquensis*	Enveloped, spherical/~100 nm	Tripartite, linear ssRNA (−)	~6.1 kb (L), ~1.2 kb (M) and ~0.7 kb (S)	ORF1, CP and RdRp	2020
GdRV-2	*Totiviridae/Giardiavirus*	G. duodenalis	/	/	~6.5 kb	ORF(L): ~200kDa ORF(S): ~17kDa	2021
TVV5	*Totiviridae/Trichomonasvirus*	T. vaginalis	/	Monopartite, linear dsRNA	~5.1 kb	CP and CP/RdRp	2022

a In family/genus, LRV1’s family is Totiviridae and its genus is Leishmaniavirus; Similarly, in Virion (shape/number), LRV1’s shape is icosahedral and its diameter is approximately 40 nm (~ meaning approximately); In ORF, LRV1 has two ORFs, CP and a fused form of CP with RdRp; In blank sections, such as Genome, Genome size and ORFs for Entamoeba histolytica virus, only a “/” is contained, the “/” meaning that this section is not currently being studied. The same applies to other cases.

**TABLE 2:** Summary of the main characteristics of the viruses found in *Eimeria spp*

Virus	Pathogen	Virion (shape/diam)	Genome	Genome size	ORFs	Earliest discovery time
EsRV1	*E. stiedae*	Icosahedral/~ 35 nm	Monopartite, linear dsRNA	~6.2 kb	CP and RdRp	1989
ENV1	*E. nieschulzi*	unidentified/~ 39 nm	Five-segmented, linear dsRNA	~0.57 kb, ~0.72 kb, ~5.0 kb, ~5.7 kb, and ~11.5 kb	CP and RdRp	1994
*E. acervuline virus*	*E. acervulina*	Icosahedral/42-44 nm	Bi-segmented, linear dsRNA	~1.7 kb and ~3.5 kb	CP and RdRp	1996
*E. maxima virus*	*E. maxima*	Icosahedral/42-44 nm	Monopartite, linear dsRNA	~4.5 kb	CP and RdRp	1996
*E. necatrix virus*	*E. necatrix*	Icosahedral/42-44 nm	Bi-segmented, linear dsRNA and ssRNA	~4.2 kb (double-stranded) and ~5.6 kb (single-stranded)	CP, RdRp and transcriptase	1996
*E. brunetti virus*	*E. brunetti*	Icosahedral/42-44 nm	Bi-segmented, linear dsRNA	~2.1 kb and ~3.3 kb	CP and RdRp	1996
EtRV1	E. tenella	Icosahedral/~ 38 nm	Tripartite, linear dsRNA	~3.6 kb (L), ~2.4 kb (M) and ~1.4 kb (S)	CP and RdRp	2011

## PARASITIC PROTOZOAN: STRUCTURE AND LIFE CYCLES

Recent studies have investigated the structure and life cycle of different PPVs, which differ based on their structure, host species, and infection routes and show significant diversity and specificity.

### Giardiavirus.

GLV has an unusual internal ribosomal entry site (IRES) that drives viral protein translation (30). Studies have shown that the single-stranded copy of the viral genome serves a dual function as an mRNA and a replication vector (31). The IRES contains a pseudoknot U3 and a set of stem-loops located in the 5′-untranslated region (UTR) that cover the upstream and downstream regions of the start codon. The upstream encoding domain of the start codon includes three U4 units and U5, and the downstream region contains stem-loop I, the downstream box (DB), and another pseudoknot (32). Two pseudoknots participate in transcript stability maintenance and the U4 protein in the translation process. The small ribosomal subunit localization is affected by U5, stem-loop I, the AUG start codon and the distance between them (30, 33). Notably, stem-loop I, the DB sequence and pseudoknots delay ribosome movement during translation and allow error-corrected identification of the start codon (34, 35), which contributes to CP and RdRp expression (36).

To attract small ribosomal subunits for initiating translation, GLV must bind to a variety of proteins, such as SRp20 and IRES-binding protein 1 (IBP1) (37). Additionally, Giardia-specific cysteine proteases remove N-terminal 32 amino acids during post-translational processing, which facilitates viral protein maturation (38). These 32 amino acids are hypothesized to represent the GLV membrane-permeable peptide. All 32 amino acids are tiny and only 2 are charged, which facilitates viral entry (39). Stem-loops and the last 37 nucleotides at the 3′ end of GLV ssRNA, which mark the site of commencement of replication, are essential for the GLV genome (40, 41). The *Totiviridae* family members conceal dsRNA in the host cytoplasm by enclosing the positive RNA strand into subviral particles, using RNA polymerase to create the negative strand, followed by a combination of 2 ssRNAs based on the complementary base pairing principle (42).

The GLV genome plays a crucial role in the life cycle of GLV owing to its unique function and structure. *Giardiavirus* transcripts electroporated into Giardia trophozoites can produce mature, contagious viral particles (43). Similarly, Giardia trophozoites co-incubated with purified GLV particles produce infective GLV progeny (43, 44). Notably, GLV does not separate from Giardia by cell lysis and subsequently infects other virus-free Giardia (7). GLV specificity is highlighted by its inability to infect other species of protozoan parasites (45) or to induce pathological changes in mammalian intestinal cell lines, similar to this property observed in other *Totiviridae* viruses (46).

The mechanism of Giardia infection by GLV remains unclear. Reportedly, GLV enters trophozoites through receptor-mediated endocytosis because it can be prevented by specific inhibitors (47), although this inhibitory pathway is not well established. Wang et al. (46) prepared polyclonal antibodies against the CP, which inhibited virus entry into Giardia to prove CP’s involvement in virus invasion. However, the viral receptor remains unidentified, and its ligand may be an epitope of CP (46). GLV has no surface-projecting fibers that can bind to receptors. The three-dimensional GLV structure shows uncharged residues in the folded CP that may facilitate virus entry (39). An endocytosed virus is concentrated in peripheral vesicles, and low pH aids with the release of nucleic acids (47). The replication process is slow during early-stage GLV infection and rapidly increases exponentially, subsequently. Intranuclear virions were observed after exponential viral growth (48). Interestingly, not all Giardia isolates are susceptible to GLV infections (44). GLV was only observed in the WB strain when purified GLV virus particles were used to infect the WB, Ac, and JH strains, which suggests that resistant strains may be secondary to Giardia’s altered or absent surface receptors (44, 48).

### Leishmaniavirus.

The LRV contains 3 ORF segments; ORF2 encodes the viral CP, a sequence that spontaneously assembles into VLPs when expressed *in vitro*, and ORF3 encodes the viral RdRp, which contains 6 conserved consensus RdRp motifs. RdRp has a 71 nt overlap with the CP structural domain, which suggests that viral RdRp is expressed via ribosomal frameshifting (49). The CP-polymerase polyprotein of the LRV is similar to that of other *Totiviridae* family members. ORF1 and ORF X located in the 5′-UTR encode proteins without homology to any of the identified protein sequences. Interestingly, all known LRVs share > 90% nucleotide similarity with ORF1 and ORF X, which are specific fragments of LRV1-4 (50).

Using a modeling approach to estimate the LRV replication cycle, we hypothesized that the viral life cycle has the following characteristics: after transcription of dsRNA from mature viral particles, the transcription product is extruded from the capsid (51). Viral proteins are produced using IRES translation within the 5′-UTR, without the involvement of the capsid and low molar abundance of viral particles. As viral abundance increases, capsid endonuclease cleaves the viral transcript, which slows translation progress (51). The cleaved 5′-UTR and 3′-cleaved products compete for host proteins simultaneously, which may affect viral translation. A CP-polymerase polyprotein, which is less abundant than CP, is also translated. Polyprotein cleavage by the host-encoded cysteine protease is a critical step in the regulation of viral copy numbers or viral protein generation (52). The encoded RdRp binds to the viral full-length RNA and reorganizes the new dsRNA genome through positive-negative strand binding.

Other *Totiviridae* family members can spread vertically and/or horizontally (53). Armstrong et al. transiently electroporated LRV1 into LRV-free strains (54). Another interesting discovery was of *L. guyanensis*-induced exosomes that carry > 30% of virus particles. Through exosome encapsulation, LRV1 can resist an unfavorable external environment and infect other parasites (55). However, virus-infected and -uninfected strains were identified in the same culture, which suggests resistance to viral invasion in *Leishmania* spp. Additionally, mature viral particles can be transmitted to the next parasite via cell division (56, 57).

### Trichomonasvirus.

The positive strand of each TVV encodes 2 ORFs, CP and RdRp (58, 59). The overlap between these 2 ORFs is approximately 16 to 123 nt. CP/RdRp is expressed as a fusion protein through the +1 (TVV1) or -1 (TVV2, TVV3) ribosomal frameshift, which is similar to the GLV genome (60–62). Secondary structure predictions for the 5′ and 3′ ends of the TVV1 positive strand suggest a large 3′ but not a similar 5′ stem-loop (60, 61). In contrast, TVV2 has a stem-loop at both ends of the positive strand (58). This secondary structure may serve as a signal for RNA replication and/or packaging (12). Asymmetric positive strand transcripts are initially produced in TVV by RNA transcription, followed by the production of full-length transcripts by mimicking the positive strand in an end-to-end pattern. The single-stranded negative strand is synthesized within viral particles, and 2 single strands combine to form a newly assembled viral genome (12). TVV2 contains 60 copies of icosahedral asymmetric units (2 dimers of capsid protein, CP-A, and CP-B). TVV2 shows only lateral CP interlocking, which is consistent with an assembly strategy that allows TVVs to remain within the intracellular replication cycle in T. vaginalis (63). The atomic structure reveals that the predominantly negative charge in the interior of the capsid facilitates movement of the loose genome, and the 5-fold vertices are considered mRNA release pathways (63). The conserved helix-rich fold within the TVV2 CP and putative guanylyltransferase domains along the capsid exterior may play crucial roles in mRNA maintenance in the *Totiviridae* genus (63).

Research on TVV infection and the association of TVV with organelles is currently limited. Some studies have described the subcellular localization of TVV replication and assembly complexes (12). Viral proteins and VLPs were identified near the plasma membrane and Golgi apparatus in the T. vaginalis cytoplasm, and viral particles were detected in vacuoles or within endocytic-coated pits, however, the significance of these findings remains unclear (64, 65). TVV undergoes asexual proliferation (binary fission) (66) for self-replication and division, which facilitates its vertical transmission in T. vaginalis (12), which may also explain its inability to infect new trichomonads. Some TVV-infected T. vaginalis strains have dsRNA molecules that range in size from 0.5 to 1.7 kb (13, 61, 67). These molecules may serve as templates for viral RdRp and can be packaged into capsids independent of genomic dsRNA (13, 68). Moreover, as satellite dsRNAs, they are unable to encode RdRp (14) and have low homology with the TVV genome (68). Although the biological significance of these satellite dsRNAs remains unclear, they help TVV to infect T. vaginalis, similar to other *Totiviridae* families (12).

### Other protozoan viruses.

The *Cryspovirus* genome is relatively conserved and stable. Phylogenetic analysis has revealed that viral exchange between different host isolates may be infrequent (69–71). The 5′-non-translated regions (NTRs) of dsRNA1 are of the same length and remain intact (8, 67), however, the 3′-NTRs of dsRNA1 and the NTRs of dsRNA2 show amino acid truncations (71). Notably, multiple shared conserved positive strand sequences are observed in the dsRNA1 and dsRNA2 of the isolated CSpV1-Iowa strain (72). These regions are also fully or partially conserved in other isolates. This conservation may affect specific functions such as the packaging of nascent particles, RdRp binding, or translation (73). CSpV1 can be vertically transmitted in host cells via asexual and sexual proliferation. CSpV1 spreads within C. parvum oocysts and produces large amounts of the virus (1). CSpV1 may be released into the culture medium during early-stage C. parvum infection (74). The entire CSpV1 life cycle is difficult to observe owing to the lack of effective *in vitro* culture techniques for C. parvum.

*Eimeriavirus* structures are more complicated, with VLPs ranging from 30 to 38 nm in diameter (75). However, the number and length of dsRNA fragments are significantly different. For example, 5 different dsRNA fragments were detected in *Eimeria nieschulzi* (75), and 3 different viral bands in E. tenella (76). Wu et al. (77) sequenced the complete genome of E. tenella RNA virus 1 (EtRV1) and observed no similarity with other dsRNA viruses based on the UTR structure. As a member of the *Totiviridae* family, the complete EtRV1 genome expresses a putative CP and RdRp (78). The A base in the 5 overlapping nucleotides (URAUG, R = A or G) linking the 2 ORFs is both the last base of the stop codon of the former ORF and the first nucleotide of the start codon of the latter ORF (79). The *E. stiedae* genome contains 2 ORFs with a four-base transition fragment (AUGA) at the junction (80); the amino acid sequence is highly homologous to that of EtRV1 (80). Currently, limited data are available regarding *Eimeriavirus* biology. Virus-infected strains displayed no abnormalities or death. Interestingly, viral CP is expressed only in sporulating phages (77). These viruses are unable to infect new strains and quantification of viral particles is challenging (77).

In contrast to the life cycles of other common viruses, details regarding PPV attachment, invasion, replication, assembly, egression, and release remain still poorly understood. Although limited by PPV host specificity, further studies are warranted to investigate their structure and life cycles as more viruses continue to be discovered.

## PARASITIC PROTOZOAN VIRUS-MEDIATED MULTIPLE REGULATIONS

Recent studies have investigated the association between protozoan viruses, parasites, and hosts, which will provide a greater understanding of the cycles of viral infection and the feedback effects of host immune responses.

## REGULATION OF THE T. VAGINALIS VIRUS

TVV was the first virus isolated from a protozoan parasite; the interrelationship between TVV and T. vaginalis has become increasingly important. Wang et al. determined that the initial phenotypes of T. vaginalis isolates were correlated with dsRNA (81). Freshly isolated strains contain viral particles, and long-term *in vitro* cultures show a completely negative TVV phenotype, which suggests that viral dsRNA loss (81, 82) was consistent with the absence of surface immunogens and the loss of phenotypic changes in T. vaginalis (83, 84). In addition to T. vaginalis phenotype alternations, TVV causes severe cytopathological changes in TVV-infected cells (64, 85). TVV transfection led to the lysis of many T. vaginalis with the recruitment of reminiscent cells to form large clusters. Few viral particles were detected in the nucleus, predominantly in the cytoplasmic vacuoles and in coated pits (85). The residual membranes of lysed cells were lined with the virus. The Golgi complex showed changes in electron density and cisternae structure secondary to virus activity (64).

Endosymbiotic TVV was not cytotoxic to T. vaginalis and might be beneficial to its adaptation and virulence in the human host (86). Similar to other protozoan viruses, TVV may not replicate in human host cells, yet the TVV genome or gene products are shed from T. vaginalis, which triggers an inflammatory response during T. vaginalis infection and/or conventional antibiotic treatment (87). Fichorova et al. reported that TVV-infected isolates induced interferon-β (IFN-β) and triggered an elevated pro-inflammatory response compared with TVV-free strains. TVV-infected strains depend on the inflammatory response induced by endosomal acidification, a process accomplished through Toll-like receptor-3 (TLR3) upregulation (88, 89). A significant concern in the medical community was that treatment of TVV-infected strains using antibiotics such as metronidazole increases the risk of inflammation-associated reproductive diseases. In the absence of cytotoxicity, infection of human endocervical cells with purified TVV1 or metronidazole-treated TVV-infected strains increases TIR-domain-containing adaptor-inducing interferon-β (TRIF)-mediated interleukin (IL)-1β, IL-6, IL-8, IFN-β (90), and RANTES (91), as well as decrease anti-inflammatory IL-1RA levels (90), which increases the risk of vaginal diseases and the transmission burden. TVV-induced amplification of the inflammatory response is a double-edged sword that may contribute to the clearance of some pathogens (87) but may result in other cytokine-induced recruitment of pro-inflammatory cells to the inflammation sites and promote ongoing inflammatory injury. Nuclear factor- κB (NF-κB) activation and expression of some cytokines drive long-term self-replication of pathogens (92).

However, this finding did not explain whether TVV infection affects the resistance of T. vaginalis to metronidazole (87). Ding et al. (9) observed that alcohol dehydrogenase-1, hydrogenosomal oxygen reductase and thioredoxin reductase were significantly downregulated in TVV-infected strains compared with their levels following exposure to TVV-free strains. L-lactate dehydrogenase expression was significantly upregulated in TVV-free strains (9), which may explain the greater resistance of TVV-free strains compared with that of TVV-infected strains. However, upregulation of the human ATP-binding cassette (ABC) transporter family proteins in TVV-infected strains (93) (which is involved in drug resistance in a variety of pathogenic protozoa) (94) leads to confusion regarding the regulation of drug resistance by TVV to T. vaginalis. In addition to its effect on drug resistance, TVV has been implicated in the overexpression of the AP33-1 and AP51-3 adhesion proteins (95, 96), which are associated with the pathogenicity of T. vaginalis. Some proteins, such as the ABC EI protein, heat shock proteins (translation and translocation) (97) and EF-1α (RNA polymerase binding), are affected by TVV, which indicates that TVV affected T. vaginalis gene transcription and protein expression (9).

Recent studies have discovered the role of endosymbiotic viruses in protozoan extracellular vesicles (EVs) in host immunity (98, 99). Govender et al. (99) observed that human vaginal epithelial cells stimulated by small extracellular vesicles (sEVs) released by TVV-free strains undergo significant NF-κB activation with elevated IL-8 and RANTES levels compared with the sEVs of TVV-infected strains. Notably, sEVs of virus-positive parasites show immunosuppressive effects (99). These data support the possibility that T. vaginalis-TVV symbiosis may contribute to immune evasion by T. vaginalis using sEVs as carriers of intercellular communication and protein modification to inhibit host immune activation (99).

## REGULATION OF THE *LEISHMANIA RNA VIRUS*

Similar to findings in TVV, purified LRV is unable to infect the parasite (100) and must utilize alternative pathways to invade the *Leishmania* spp. The presence of 2 LRV1 dsRNAs (naked LRV1 and encapsulated LRV1) particle clusters in the *Leishmania* exosome envelope (55) suggests that LRV1 is encapsulated within *Leishmania* via the exosomal pathway rather than being encapsulated by the plasma membrane. This serves as the underlying mechanism that protects LRV from extracellular enzymes or the immune system. LRV1 affects exosomal protein content (55). For example, levels of cyclophilin A, a key factor in viral replication, are elevated in exosomes, and polysome profile analysis further demonstrates its role in the LRV1 life cycle (101). Virus-containing exosomes aggravate leishmaniasis progression and naked viruses do not cause exacerbation, which is attributable to the fact that exosome encapsulation enables LRV1 recognition by the host (55).

LRV can regulate *Leishmania* spp in a variety of regulatory ways. Widmer et al. observed that the CP can self-assemble into the virus-like capsid in LRV-free strains (102), and that capsid overexpression in naturally infected LRV-infected strains led to progressive LRV copy number reduction. These findings suggest that CP may impede LRV replication, and that parasite viability is unaffected by reduced virus copy numbers (102). Based on the proteomic and multi-genomic analysis, translation initiation and efficiency of specific mRNAs were reduced in LRV-infected parasites. The translation of some proteins, such as HSP70, GP63, and cyclophilin A was also affected (55, 103). Interestingly, significant amounts of small RNAs from LRV1 were detected in *L. braziliensis* and *L. guyanensis*, which have the same properties as the cleavage function of Dicer. The cleavage function of small RNAs and LRV1 replication maintains the dynamic LRV balance (104). Furthermore, LRV can also use miRNAs or inflammatory cytokines to maintain parasites in macrophages and enhance macrophage survival (105, 106). Reportedly, miR-155 was strongly upregulated in macrophages infected with LRV1-infected *L. guyanensis*. LRV1 can enhance macrophage survival in a miR-155-dependent manner via AKT activation, thereby promoting parasite persistence (105).

In addition to the regulation of *Leishmania*, LRV also affects the host-parasite interaction. LRV1 is associated with TLR3/TRIF-mediated production of high cytokine and chemokine levels in *L. guyanensis*-infected macrophages (107, 108). Similarly, LRV2-infected macrophages depend on TLR3 for the release of high levels of cytokines (109). Infection of human and mouse macrophages with LRV-infected *Leishmania* induces ATG5-mediated cellular autophagy by triggering the TLR3/TRIF pathway and promoting type I IFN production (10). This process downregulates the NLR family pyrin domain containing 3 (NLRP3) and apoptosis-associated speck-like protein containing a caspase recruitment domain (ASC) expression, which limits inflammasome activation within macrophages. The LRV-activated signaling pathways reduce inflammasome activation; however, they do not affect parasite replication and consequently exacerbate the extent of lesions in patients showing LRV-infected strains. LRV also inhibits caspase-11 activation and IL-1β release in a TLR3- and ATG5-dependent manner and interferes with inflammasome activation (110). Interspecies disparities also affect LRV-mediated immune effects. In a mouse model, deletion of both TLR9 and MyD88 promoted lesion progression and increased levels of Th2-associated cytokines (IL-4 and IL-13) in leishmaniasis (111), which suggests that TLR9 and MyD88 play crucial roles in the Th1-mediated healing response against *L. guyanensis*. The pro-inflammatory biomarkers’ expression in *L.* (*v*)*. braziliensis*-infected human macrophages was significantly downregulated compared with that in LRV1-free strains, which elicited a Th2-biased immune response (112), which may be attributable to the species-specific effect of LRV1 on American tegumentary leishmaniasis (ATL) (112).

Research has focused on the potential association between LRV and disease manifestations. LRV is more frequent in mucosal than in cutaneous lesions (113) and is reported in all clinical presentations, with a higher prevalence in metastatic (mucosal leishmaniasis and disseminated leishmaniasis/diffuse cutaneous leishmaniasis) than in non-metastatic forms (cutaneous leishmaniasis) (108, 114). However, no study identified has observed a direct association between clinical phenomenology and LRV1 (115). LRV is known to aggravate the pathogenic potential of leishmaniasis (111, 116), which is inextricably linked to the treatment failure of several cases of leishmaniasis (117, 118). Some drugs, such as pentamidine (119, 120), and glucantime (121), are ineffective against LRV-infected *Leishmania* infections (117, 118), and some patients develop complex lesions and persistent infections. Therefore, LRV1 may predict treatment failure and symptom recurrence and may serve as a guide for the treatment of leishmaniasis.

## REGULATION OF THE G. LAMBLIA VIRUS

Unlike LRV and TVV, purified GLV can directly infect Giardia trophozoites without utilizing the exosomal pathway (47). Owing to this difference, GLV provides the possibility of studying the symbiotic eukaryote-virus association. Infection of G. lamblia with low titers of GLV did not affect its growth. When the titer reaches approximately 5 × 10^5^ viruses/trophozoite, the growth of Giardia ceased and changed from an attached to a suspended state, which is referred to as “pseudo-death” which may be attributable to the involvement of miRNA1 in GLV, which regulates the virus copy numbers (122). It is unclear whether GLV can survive Giardia's transition from trophozoites to cysts (39, 86). An early review did not find a correlation between GLV and Giardia virulence (48). Currently, the GLV effects on Giardia infectivity remain unknown.

Previous studies have discussed the role of GLV in the innate immune response of Giardia to host cells. Li et al. showed that GLV-infected G. lamblia activated host TLR3 and its downstream NF-κB signaling pathway, which led to increased secretion of TLR3-dependent pro-inflammatory cytokines (tumor necrosis factor-α, IL-6 and IL-12), which enhances host resistance to GLV-infected G. lamblia (unpublished data). This is similar to the LRV genome (123) and may be secondary to the recognition of the GLV genome by the host cell endosomal TLR3. Xudong et al. found that both GLV-free and GLV-infected Giardia trophozoites significantly increased TLR9 expression in peritoneal macrophages obtained from wild-type mice (124). Activated TLR9 modulates host-secreted cytokines during early infection, and acts as a protector against the host. These findings support the innate immune response to GLV-regulated Giardia infection observed in host cells and provide potential targets for novel therapeutic strategies against G. lamblia.

GLV is not only involved in the regulation of the innate immune response of G. lamblia to the host but is also a very suitable transfection vector for studying the genetic constitution of Giardia (125, 126). The luciferase gene was linked to the UTR flanking the GLV genome, followed by the transfection of G. lamblia trophozoites (40, 41) with the construction of a chimeric virus that enabled luciferase protein expression. This suggests that GLV may serve as an engineered virus for heterologous protein expression in eukaryotes, which provides new pathways to expand opportunities for functional analysis of other Giardia genes.

## REGULATION OF OTHER PROTOZOAN VIRUSES

It is unknown whether CSpV1 affects parasite virulence. Although the relationship between viral load and parasite proliferation remains unclear (127), a study has shown that the number of oocysts produced by the parasite was proportional to the viral load in cell infection experiments using 2 different *Cryptosporidium* strains (with viral counts at different levels). This parasite can survive and reproduce extracellularly for a short period even without a host (128). There are no studies discussing the role of CSpV1 on the host’s innate immune response, but studies have reported this finding in *Eimeriavirus*. Lixiao et al. found that infection with virus-containing strains elicits an earlier immune response than infection with virus-free strains (129). Changes in TLR3, TLR7, and TLR21 may be attributable to the innate immune response to E. tenella in chickens. Notably, IL-6 and IFN-β production was higher in virus-containing strains than in virus-free strains accompanied by more severe pathological changes (129), which may be due to differences in pathogenic mechanisms associated with virus-containing and virus-free strains. Viral vectors for both CSpV1 (67) and *Eimeriavirus* (79) were successfully constructed and expressed heterologous proteins (such as the green fluorescent protein) in oocysts. The establishment of viral vectors provides a new perspective on gene expression modulation in parasitic protozoa.

PPVs also have detrimental effects on protozoa, such as Acanthamoeba polyphaga mimivirus (APMV), a dsDNA virus, which includes four family members (5). The virus uses protozoa as vectors for reproduction. APMV triggers the *Acanthamoeba* lysis, however, cyst formation is unaffected. This process is involved in APMV interference with serine protease expression (11).

## CURRENT ISSUES AND FUTURE CHALLENGES

Current research in protozoa is focused on the pathogenicity of parasitic protozoans to the host and immune response modulation. Among the several factors that influence parasite pathogenicity and invasiveness, PPVs are undoubtedly the most specific. However, the exact mechanism underlying PPV-induced infection in parasitic protozoa remains unclear. It is hypothesized that PPVs are residual nucleic acid fragments from organelle degradation during protozoan evolution or that exogenous genes enter the protozoan and co-evolve to preserve them (21). Although uncertain, these mechanisms may contribute to PPV function and regulation. The structure and morphology of PPVs, a generic term for a range of viruses from different families and genera, are closely associated with the viral life cycle. Although most PPVs are monomeric linear dsRNA genomes (130, 131), differences in infecting species have led to the development of various forms as follows: LmarLBV1 is a tripartite linear ssRNA(−) (22), CSpV1 is a bi-fragmented linear dsRNA and MaRNAV1 is a bi-fragmented linear ssRNA(+) (1). Information diversity affecting the genome may be attributed to the variability of parasite species. For example, LmarLBV1 is currently only found in *Leishbunyavirus* (22) and TVV only in T. vaginalis. Moreover, other segments of the genome can impact the life cycle of protozoan viruses; however, the specific biological significance of satellite dsRNAs in the TVV genome is unknown (14), although these may participate in TVV replication and assembly (13). In summary, diversity in PPV structure and genomes results in a lack of clarity regarding the viral life cycle, which is challenging for investigating viruses that infect eukaryotes.

Notably, PPVs are not similar to common mammalian viruses in the regulation of parasites and hosts. Theoretically, PPVs require protozoa for survival and derive important components from protozoa. In contrast to common mammalian viruses, PPVs, such as GLV, do not cause host cell lysis (7). When the GLV copy number increases to a threshold value, Giardia enters a “pseudo-death” state (122). At this stage, the biological activity mechanism in Giardia itself and those associated with the completion of the GLV life cycle remain unknown. Furthermore, receptors associated with PPVs that infect protozoa are unclear. Most PPVs lack the explicit viral receptor binding structure due to the smoothness of their surface, making investigation of PPV invasion difficult (39). Research has suggested that the hydrophobic region of the CP surface mediates viral invasion of host cells. There is a lack of knowledge regarding protozoan proteins and organelles used by PPVs to complete their life cycles, including self-replication and assembly mechanisms. Genomic minimization of lower eukaryotes, such as Giardia and T. vaginalis simplifies protozoan proteins (132); therefore, proteins required for the replication and assembly of PPVs become less complicated. This finding has important implications for the study of eukaryotic-viral coexistence. It would be interesting to investigate whether PPV infection causes protozoan proteins and organelles to contribute to viral resistance.

PPVs affect hosts through complicated biological processes. They can exacerbate the host inflammatory response via activation of host cell TLR3 (87), alterations in the infective process, and immunopathological changes that promote parasite survival (10, 100). However, protozoan infection and their pathogenesis are complex. For example, LRV-infected strains can cause severe pathological changes in leishmaniasis compared to LRV-free strains (120, 121); there is also an opposite trend. Currently, there are no studies that have explicitly reported the factors associated with successful or failed treatments of protozoan diseases. PPVs can inhibit the innate immune response of host cells through EVs, slowing inflammasome activation and cytokine production, all of which favor immune evasion by protozoa (55, 133). Therefore, PPVs prolong their survival via host metabolism regulation and slowing host immune system clearance. These multi-directional and multiple regulatory mechanisms provide an important theoretical basis for the study of eukaryotic virus-host interactions and antiviral therapy development.

## CONCLUSION

A deeper investigation of the various processes that include PPV involvement, such as protein production and activity during infection, membrane fusion during internalization, viral replication and assembly, and the mechanisms by which hosts evade viral infection or whether PPV can infect protozoa at different protozoan periods can contribute to understanding the regulatory mechanisms of PPVs. As described earlier, researchers have investigated the use of these viruses as tools to study parasites. Following the widespread detection of drug-resistant strains, these findings can guide the treatment of protozoan infection.
